# Incidence of NUT carcinoma in Western Australia from 1989 to 2014: a review of pediatric and adolescent cases from Perth Children’s Hospital

**DOI:** 10.1186/s12885-021-08432-0

**Published:** 2021-06-27

**Authors:** Tina Carter, Maxine Crook, Ashleigh Murch, Alex H. Beesley, Nick de Klerk, Adrian Charles, Ursula R. Kees

**Affiliations:** 1 Perth Children’s Hospital, Perth, 6009 Australia; 2 PathWest, Nedlands, 6009 Perth, Western Australia; 3 Telethon Kids Institute, University of Western Australia, Perth, 6009 Australia; 4 Department of Pathology, Sidra Medical and Research Center, PO Box 26999, Doha, Qatar

**Keywords:** NC incidence, Carcinoma, Undifferentiated malignancy, NUTM1, Heterogeneity, Diagnosis, Rare, Aggressive

## Abstract

**Background:**

NUT carcinoma (NC), previously known as NUT midline carcinoma, is a rare and very aggressive cancer that occurs in both children and adults. NC is largely chemoresistant, with an overall survival of less than 7 months. Because the carcinoma is not restricted to a particular organ, diagnosis is often a challenge. In the absence of a clearly determined incidence for NC, we sought to study the diagnosis of patients in a well-defined population.

**Methods:**

We systematically reviewed records of all patients that presented to the Oncology Department of the Princess Margaret Hospital for Children from 1989 to 2014. This institution in the geographically isolated state of Western Australia has a catchment population of around 2 million. We then identified all high grade undifferentiated sarcomas or carcinomas in the 0–16 year age group.

**Results:**

Over 26 years, we found 14 patients of 16 years or younger with undifferentiated malignant tumors. Of these, five tumors were positive by immunohistochemistry for the NUT/NUTM1 (Nuclear Protein in Testis) protein and/or the translocation t(15;19). Three patients presented with thoracic tumors, one with a para-spinal tumor, and one had an upper airway nasopharyngeal carcinoma. In all five cases, there was an initial response to therapy and then progression. This 26-year survey was conducted in a geographically isolated state with a well-defined population, and we determined an estimated incidence of NC of around 0.41 per million child years (0–16 yrs. of age) at risk. From three patients it was feasible to derive cell lines for further genetic analyses and drug screening.

**Conclusions:**

For the first time, the incidence of NC could be determined in a well-defined geographic area. The calculated rate of NC incidence is consistent with a history of under-recognition for this malignancy. These findings indicate that improved diagnostic detection of NC would enable better management and counselling of patients. Our findings emphasize the heterogeneity of NC, and they highlight the need to develop personalised therapy options, and to consider a diagnosis of NC in undifferentiated malignant tumors.

## Background

NUT carcinoma (NC), previously referred to as NUT midline carcinoma (NMC), is a rare and very aggressive cancer that occurs in both children and adults [1, 2]. NC was first described with the characteristic t(15;19) translocation almost 30 years ago [3, 4]. Since NC is rare, not restricted to a particular organ and lacks specific clinical and histomorphological features, the diagnosis is often challenging and misdiagnoses occur [2, 5, 6]. The characteristic translocation t(15;19) results in the fusion of the *NUTM1* gene (previously called *NUT*), located at 15q14, with another gene. In most cases this gene is *BRD4* at 19p13, and various translocation breakpoints have been reported [1, 7–9]. The remaining cases show translocations between *NUTM1* and non-*BRD4* genes, including *BRD3* or *NSD3,* or other genes [1, 2, 10–12]. These fusions lead to the formation of oncogenic complexes that, in cell lines, prevent squamous cell differentiation, and alter histone acetylation [11, 13–16].

These translocations appear essential to the development of NC, which is otherwise associated with an apparently simple karyotype. This type of genetic change is seen in many childhood leukemias, some lymphomas and soft tissue tumors, in contrast to the complex karyotypes and multiple genetic events seen in most carcinomas [13, 14]. NC can affect any age, from neonatal cases [1, 17] to older patients. However, many are described in young patients which, with the notable exception of nasopharyngeal carcinoma, is unusual for carcinomas.

NC is an aggressive and largely chemoresistant disease. A 2012 study reported that 63 patients who were diagnosed with NC were found to have an overrall survival of 6.7 months; the 2 year progression free survival was 9% and the overall 2 year survival was 19% (CI 7–31%) [18]. A review of the literature showed the overall survival in 119 patients to be only 5 months [19]. Occasional cases with a better outcome are reported [20, 21].

Poorly differentiated tumors requiring immunohistochemistry and/or molecular techniques for diagnosis are not uncommon in pediatric and adolescent patients. However, there remains a small residual heterogeneous group that do not clearly fit into a diagnostic category, and these have a variable prognosis and response to therapy. Tests to exclude NC and other entities such as the Rhabdoid tumor group should be routinely considered. Establishing a diagnosis of a NC tumor is important to enable better management and counselling, so we aimed to conduct a survey to identify NC tumors over a period of 26 years at the only tertiary pediatric institution of the geographically isolated state of Western Australia.

## Methods

We reviewed records from the Pathology and Oncology Department of all patients that presented to the Princess Margaret Hospital for Children Oncology Department from 1989 to 2014 with an undifferentiated or poorly differentiated malignancy. In 2018, the hospital changed name to Perth Children’s Hospital (PCH). The Oncology-Haematology Department at PCH is a member of the North American Children’s Oncology Group (COG), and all patients are invited to participate in COG studies (where these are open) or are treated according to previous COG protocols. Tumor specimens were assessed using formalin or B5-fixed material, routine histology and immunohistochemistry, including hematoxylin and eosin (H&E), cytokeratin AE1&3, vimentin, CD99, INI, neuron-specific enolase (NSE) [22, 23]. The NUTM1 protein was detected using the C52B1 antibody (Cell Signaling Technology, USA). Microscopy images were acquired using Leica equipment for routine diagnostic microscopy and image acquisition. Samples were taken for cytogenetics and molecular genetic analyses. To select the cases as potential NC for this review, we first identified all patients with an undifferentiated or poorly differentiated malignancy seen at PCH over the 26-year period. Of those, the histology, immunohistochemistry, cytogenetics and site of tumor allowed diagnosis of most cases. Special consideration was given to cases where an NC was possible, such as poorly differentiated carcinoma in or around the respiratory tract, or where cytogenetic results on review were suggestive of NC. From the mid 1990’s, additional targeted molecular tests such as FISH became available clinically, although NUT FISH was not available clinically until 2013. Some of the undifferentiated or poorly differentiated malignancies were found to be newer and previously undescribed entities. Based on the review of all patients with an undifferentiated or poorly differentiated malignancy at PCH over a 26-year period, fourteen cases remained difficult to classify. At the time of diagnosis, patients or their guardians consented to providing samples for research, including attempts to establish cell lines from tumor material. Ethical approval for the study was obtained from the Human Research Ethics Committee of the Princess Margaret Hospital for Children. PCH is the only hospital in the state of Western Australia that routinely treats pediatric and adolescent oncology patients aged 0–16 years. The geography and demographics of Western Australia with a largely urban population in a large state, at very long distances from other urban centers, is an advantage for epidemiological studies. We examined the incidence of this tumor and the main pediatric tumors over 26 years in Western Australia, using denominator data from the Australian Bureau of Statistics (database accessed April 2020) [24].

## Results

### Patients

From 1989 to 2014, fourteen cases presenting to PCH were identified as undifferentiated sarcoma or carcinoma (Table 1). The cases were reviewed with respect to karyotype, histopathology (including immunohistochemistry) and further immunohistochemical staining (for INI and NUTM1 where tissue was available) to determine consistency with NC. Five cases were positively identified as being NC (summarised in Table 2). The remaining cases included two confirmed nasopharyngeal carcinomas (NPCs), three rhabdoid or probable rhabdoid tumors, one INI-negative undifferentiated sarcoma, one malignant solid-type pleuropulmonary blastoma (PPB), and one malignant myxoid undifferentiated tumor (a suspected malignant peripheral nerve sheath tumor, MPNST). One tumor could not be further characterised due to lack of suitably-fixed tissue for immunohistochemistry.

**Table 1:** Fourteen poorly differentiated or undifferentiated cancers identified from hospital pathology records over 26 years

Patient Case	Age at diagnosis	Gender	Pathology	Tumor site (primary)	Chemo Therapy^a^	Radio therapy^b^	NUTM1 staining	Diagnosis	Alive	Time to death
1	13 yrs. 9 mo	M	Undifferentiated CA	Nasopharynx	CP	Y (C)	Neg	NPC	Yes	
2	11 yrs. 7 mo	F	Undifferentiated CA	Mediastinum	Ifos	Y (E)	Pos	NC	No	4.5 Mo
3	16 yrs. 7 mo	F	Undifferentiated CA	Mediastinum	CP	Y (C)	Pos	NC	No	1 yr 3 Mo
4	7 yrs. 8 mo	M	Undifferentiated CA	Larynx	CP	Y (C)	Neg^c^	NC	No	10 Mo
5	13 yrs. 6 mo	F	Carcinoma	Nasopharynx	CP	Y (E)	ND	?	Yes	
6	16 yrs. 8 Mo	F	Carcinoma	Lung	CP	Y (C)	Pos	NC	No	8 Mo
7	11 yrs. 8 Mo	M	Carcinoma	Nasopharynx	CP	Y (C)	Neg	NPC	Yes	
8	5 yrs. 2 Mo	F	Undifferentiated Sarcoma	Retroperitoneum	VAC	Y (C)	Neg	INI neg sarcoma	Yes	
9	3 yrs. 5 Mo	F	Undifferentiated Sarcoma	Neck	VAC	Y (C)	ND	Probable Rhabdoid	Yes	
10	13 yrs. 3 Mo	F	Sarcoma	Paraspinal	CP	Y (C)	ND	? MPNST	No	12 Mo
11	14 yrs. 2 Mo	F	Undifferentiated Sarcoma	Paraspinal	VAC	Y (C)	Pos	NC	No	2 yrs. 5 Mo
12	1 yr 2 Mo	M	Undifferentiated Sarcoma	Bladder	VAC	Y (C)	Neg	Rhabdoid	No	1 yr 3 Mo
13	3 yrs. 6 Mo	F	Undifferentiated Sarcoma	Lung	VAC	N	Neg	PPB	No	8 Mo
14	5 Mo	M	Undifferentiated Sarcoma	Neck	VAC	Y (C)	Neg	Probable Rhabdoid	No	6 yrs. 2 Mo

^a^Chemotherapy: (CP) Cisplatinum-based, i.e. Cisplatinum, Doxorubicin, 5FU or Carboplatinum, (Ifos) Ifosfamide, (VAC) Vincristine, Actinomycin and Cyclophosphamide

^b^Radiotherapy: (Y) yes to primary tumor or (N) no to primary tumor, (E) early with first few cycles or (C) at the end of chemotherapy to consolidate therapy

^c^Probably technical failure due to fixative

*MPNST* Malignant peripheral Nerve Sheath Tumor, *PPB* PleuroPulmonary Blastoma, *NPC* Nasopharyngeal carcinoma, *ND* Not determined, *CA* Carcinoma

**Table 2:** Pathology of NC cases and established cell lines

Patient	Tumor site	Histopathology^**a**^	Cytokeratin staining^**a**^	NUTM1 staining^**a**^	Cytogenetics of tumor^**a**^	Cell line established
2	Lung	Poorly differentiated carcinoma focal squamous differentiation	YES (focal)	YES	t(15;19)(q14;p13.1)	PER-403
3	Sternal mass	Highly necrotic small cell, poorly differentiated carcinoma, with focal epithelial differentiation	YES (focal)	YES	46 XX (probably stromal cells growth rather than tumor)	
4	Nasopharynx	Poorly differentiated carcinoma, focal abrupt squamous differentiation	YES (very focal)	NO (Technical problem)	46 XY, t(15;19) (?p11;?q12)	PER-704
6	Bronchus	Highly necrotic small cell, poorly differentiated carcinoma, with focal epithelial differentiation	YES (focal)	YES	46 XX, t(1;18;7)(q42;q11.2;q21), t(6;19)(q13;p13.1) FISH: cryptic BRD4-NUT	PER-624
11	Paraspinal L4	Small round blue cell tumor, some neural features	YES (very focal)	YES	46, XX, t(15;19)(q13;q13.3)	

^a^Tumor specimen from patient

### Incidence of NC

Over the 26 years captured by this review, there was a steady increase in the 0–16 year old population in Western Australia, from 419,412 in 1989, to 547,295 in 2014 [24]. From this we determined an estimated incidence of around 0.41 cases per million child years (0–16 years of age) at risk or 1 NC per 2.4 million child-years.

### NC case reports

#### Patient 2

**Fig. 1:**
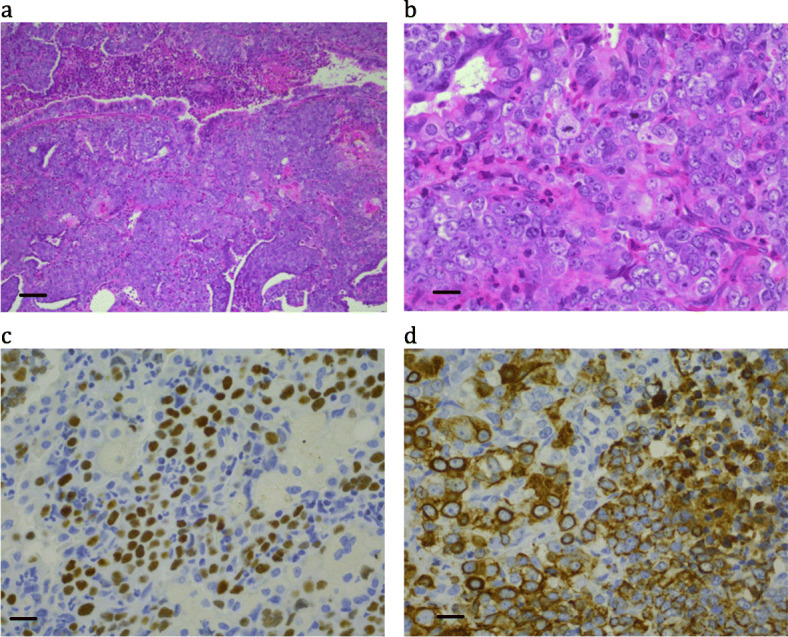
Patient 2: a poorly differentiated carcinoma with focal squamous differentiation. (a) H&E × 10; bar = 100 μm (b) H&E × 40; bar = 25 μm; (c) NUTM1 immunohistochemistry × 40; bar = 25 μm; (d) Cytokeratin AE1&3 × 40; bar = 25 μm

More than 30 years ago, an 11 year-old girl presented with a history of a cough and right shoulder pain. Imaging showed complete opacification of the right hemithorax. There was progressive enlargement of the mass leading to superior vena caval obstruction, complete replacement of the right hemithorax and displacement of the mediastinum to the left. Further investigations showed the mass was locally invasive with no distant metastatic spread. Open lung biopsy showed a poorly differentiated malignant neoplasm with focal squamous differentiation, and immunohistochemistry showed strong cytokeratin positivity (Fig. 1 and Table 2). In 1991 we reported that the intrathoracic carcinoma in this patient showed a translocation t(15;19), which at the time was a novel finding and of unknown significance [3].

Objective tumor response was noted with combined chemotherapy and radiation, consisting of 5-day courses of ifosfamide and VP16 given at 3–4 week intervals for 3 months, and radiotherapy (Table 3). After the initial tumor response, however, the tumor progressed, and 3 months later the patient presented with buttock pain, and a bone scan revealed lesions in the axial and peripheral skeleton. A course of cisplatinum with VP16 led to a brief symptomatic response, and the patient died 4.5 months after presentation.

**Table 3:** Summary of therapy to NC cases

Patient	Tumor site	Year of diagnosis	Chemotherapy	Radiotherapy
2	Lung	1989	Ifosfamide (1800 mg /m2/day) and VP16 (100 mg/m2/day)	60.4 Gy
3	Sternal mass	1996	^a^CCG 0894: carboplatinum (400 mg/m2/day), VP16 (100 mg/m2/day) and ifosfamide (1800 mg /m2/day) Autologous peripheral hematopoietic stem cell rescue with melphalan (200 mg/m2).	60.4 Gy
4	Nasopharynx	1996	^a^CCG 0894: carboplatinum (400 mg/m2/day), VP16 (100 mg/m2/day) and ifosfamide (1800 mg /m2/day)	^b^IFRT 75 Gy (supraclavicular fossa) and 52.5 Gy (posterior region of the neck)
6	Bronchus	2007	Five cycles of ifosfamide (1200 mg/m2/day) and doxorubicin (37.5 mg/m2/day) given with dexrazoxane as cardioprotectant	45 Gy
11	Paraspinal L4	1994	^a^CCG 6902, vincristine (1.5 mg/m2), actinomycin (0.15 mg/kg/day) and ifosfamide (1800 mg/m2/day) Subsequent to second debulking, cyclophosphamide (2.2 g/m2), VP16 (100 mg/m2/day). Peripheral hematopoietic stem cell rescue with melphalan (200 mg/m2).	63.8 Gy

^a^*CCG* Children’s Cancer Group

^b^*IFRT* involved field radiation therapy

#### Patient 3

**Fig. 2:**
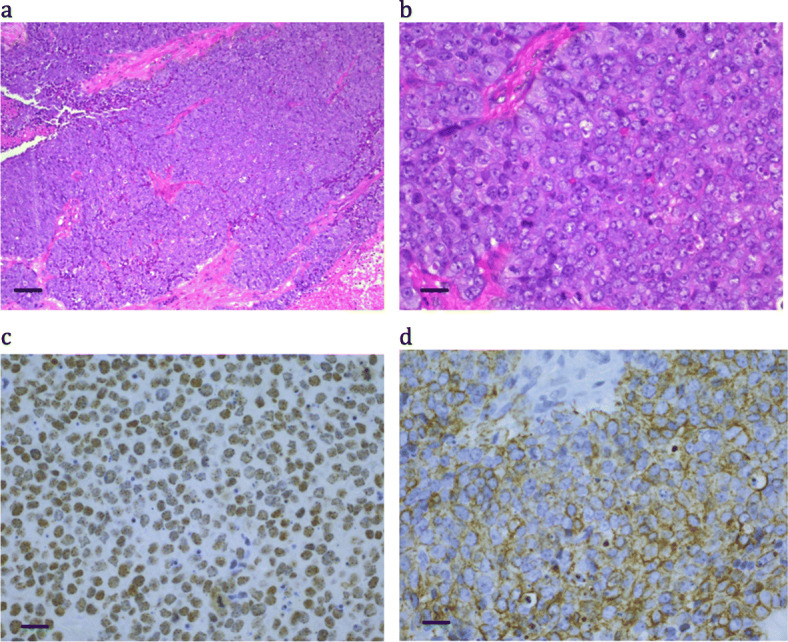
Patient 3: a highly necrotic small cell, poorly differentiated carcinoma, with focal epithelial differentiation. (a) H&E × 10; bar = 100 μm; (b) H&E × 40; bar = 25 μm; (c) NUTM1 immunohistochemistry × 40; bar = 25 μm; (d) Cytokeratin AE1&3 × 40; bar = 25 μm

A 16 year-old adolescent girl presented with shortness of breath. She had a locally invasive large mediastinal mass on imaging, which was subsequently biopsied. Further investigations did not show evidence of metastatic disease. The tumor was very poorly differentiated but showed epithelial differentiation by positive immunohistochemistry for high molecular weight cytokeratin (Fig. 2 and Table 2), but negative for low molecular weight cytokeratin and epithelial membrane antigen. Other markers including CD99, S100 and neural markers were negative. She was treated on CCG (Children’s Cancer Group) study 0894 for six cycles at 3–4 week intervals (Table 3). Local mediastinal relapse occurred 12 months after diagnosis, confirmed on biopsy, and she was treated with an autologous rescue using melphalan. She progressed and died 15 months after diagnosis. A positive NUTM1 staining was seen on review (Table 2).

#### Patient 4

A 7-year old boy presented with a 3-week history of cough, hoarse voice, noisy breathing and a left-sided neck lump. Imaging showed a left anterior irregular and discrete neck mass and a smaller similar lesion present on the right side of the neck. In addition, there was soft tissue thickening of the epiglottis and left aryepiglottic fold and a soft tissue swelling around the upper trachea was seen with some narrowing of the larynx and upper trachea. Further investigations confirmed the masses were locally invasive with some involvement of cervical lymph nodes but no distant metastatic disease was demonstrated.

**Fig. 3:**
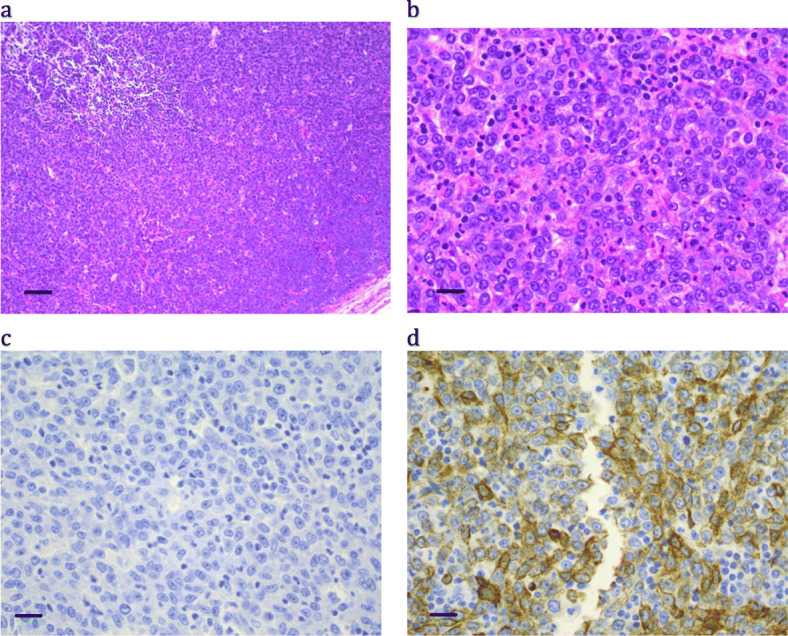
Patient 4: a poorly differentiated carcinoma with focal abrupt squamous differentiation. (a) H&E × 10; bar = 100 μm; (b) H&E × 40; bar = 25 μm; (c) NUTM1 immunohistochemistry × 40; bar = 25 μm; (d) Cytokeratin AE1&3 × 40; bar = 25 μm

Multiple biopsies showed histology of a poorly differentiated tumor with no obvious squamous differentiation (Fig. 3 and Table 2). Immunohistochemistry was equivocal for pan-cytokeratin but negative for low molecular weight cytokeratin, and was negative for CD99, neural markers and vimentin. Progression to the lymph node revealed a locally metastatic tumor with undifferentiated malignant cells, but focal abrupt transition to well differentiated squamous cells. Cytogenetics showed a t(15;19) translocation, however, on review only B5-fixed material was available, and NUTM1 was negative. Metaphases were of poor quality and a more precise breakpoint could not be determined, but since NC translocations can often be complex or cryptic [7, 9, 25], the full reported karyotype (Table 2) does not rule out a *NUTM1* translocation.

The patient was treated initially on CCG 0894 given at 3–4 week intervals (Table 3). Following two cycles of therapy, local progression was noted in the right neck and confirmed on imaging. IFRT (involved field radiation therapy) was administered (Table 3). There was resolution of the nodal masses and some resolution of the tracheal infiltration by imaging. At 3 months imaging showed new lesions in the proximal tibias and the head of the right humerus, confirmed by biopsies to be metastatic carcinoma. The patient succumbed ten months after initial diagnosis.

#### Patient 6

**Fig. 4:**
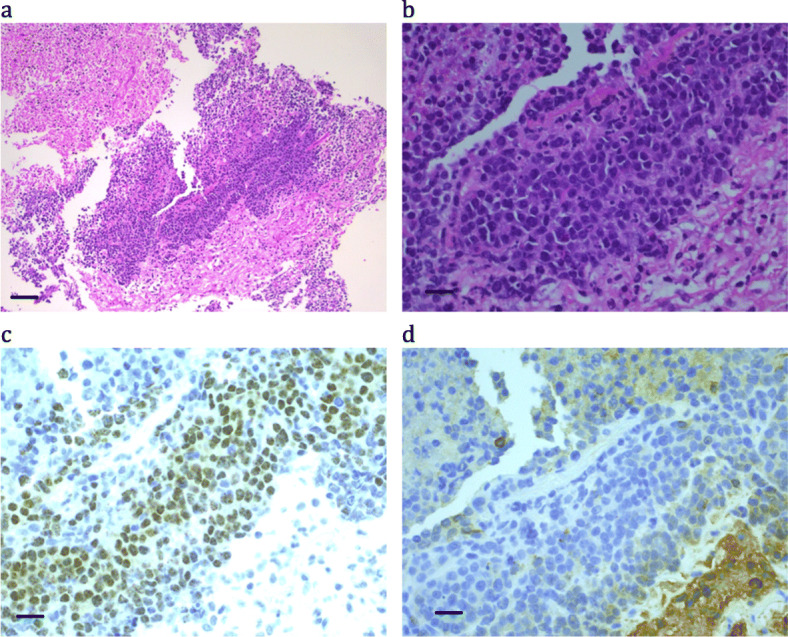
Patient 6: a highly necrotic small cell, poorly differentiated carcinoma, with focal epithelial differentiation. (a) H&E × 10; bar = 100 μm; (b) H&E × 40; bar = 25 μm; (c) NUTM1 immunohistochemistry × 40; bar = 25 μm; (d) Cytokeratin AE1&3 × 40; bar = 25 μm

A 16 year-old girl presented with a 2-week history of cough, fever and right-sided chest pain. Imaging revealed a large right-sided posterior locally invasive mediastinal mass with secondary right middle and lower lobe lung collapse/consolidation. Futher imaging did not demonstrate metastatic disease. The biopsy was very small and largely necrotic (Fig. 4 and Table 2). A diagnosis of poorly differentiated lung carcinoma, possibly NC, was made. A repeat biopsy by direct bronchoscopy showed an extensively necrotic and poorly differentiated tumor, with extensive neutrophil infiltrate, and focal squamous differentiation. The karyotype showed a complex translocation, which included the *BRD4* locus. NUTM1 positive staining was confirmed on review (Table 2).

The patient poorly tolerated 5-fluorouracil (5FU) which was part of the initial cycle of combination therapy, and developed 5FU-induced cardiotoxicity. Radiological re-evaluation demonstrated disease progression. Therapy was changed to five cycles of combination intravenous ifosfamide and doxorubicin given with dexrazoxane as cardioprotectant (Table 3), demonstrating clinical and radiological response with this chemotherapy combination. Therapeutic radiotherapy to consolidate response commenced 7 months after diagnosis, but while on radiotherapy thoracic disease recurred, and the patient died of rapidly progressive resistant disease a month later.

#### Patient 11

A 14 year-old female presented with a six-week history of right-sided anterior thigh and lower leg pain associated with difficulty of walking, pain and paraesthesia in the lumbar dermatomes 2, 3 and 4, without other associated neurological symptoms. Imaging showed a soft tissue lobulated mass at L4/5 extending through the foramina into the intradural space with some cord compression. Additional staging did not show any evidence of metastatic disease.

**Fig. 5:**
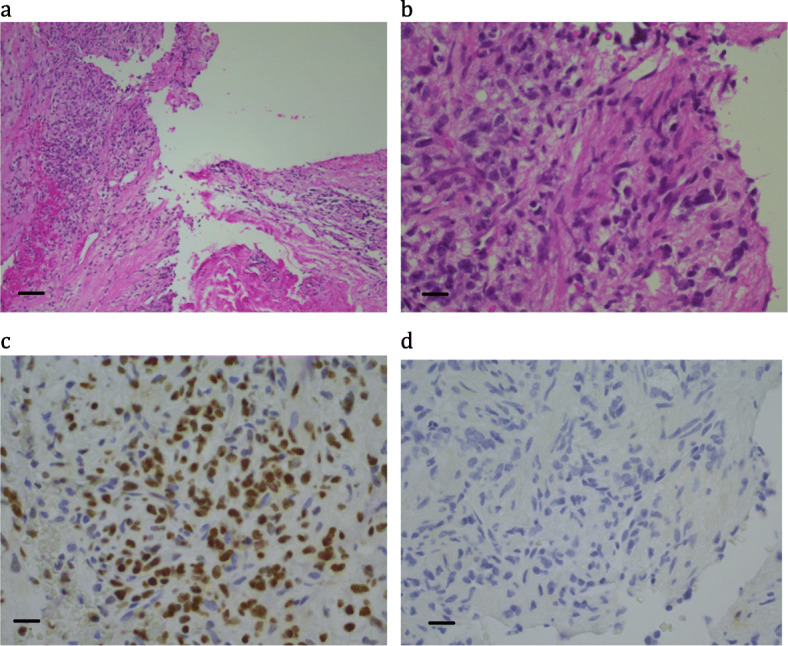
Patient 11: Small round blue cell tumor with some neural features. (a) H&E × 10; bar = 100 μm; (b) H&E × 40; bar = 25 μm; (c) NUTM1 immunohistochemistry × 40; bar = 25 μm; (d) Cytokeratin AE1&3 × 40; bar = 25 μm

Debulking surgery was performed. The tumor demonstrated sheets of poorly differentiated cells with a focally myxoid stroma and some focal cytokeratin staining, with no obvious epithelial diffentiation microscopically (Fig. 5 and Table 2). There was a hint of neural differentiation with NSE and chromogranin staining. CD99 staining was not performed. Post-surgical CT scans showed a significant residual mass in the epidural space with clearance of the intradural component. Cytogenetics revealed a t(15;19) translocation, however, the precise breakpoint could not be determined from these solid tumor preparations which only ever returned short chromosomes. Since NC translocations can often be complex or cryptic [7, 9, 25], the full reported karyotype (Table 2) is consistent with a *NUTM1* translocation, however the translocation involved the long arm of chromosome 19 and did not appear consistent with a *BRD4* rearrangement. At the time of analysis, it was considered possible that the rearrangement was more complex than was reported and that *BRD4* might still be rearranged. However, recent reports of *CIC-NUTM*1 rearrangements [26, 27] with *CIC* located on the long arm of 19 are more consistent with this patient’s karyotype and should be considered as the most likely rearrangement for this patient.

The patient was treated as per CCG protocol 6902 (Table 3). Follow-up imaging, 3 months after commencement of chemotherapy, showed little change in the size of the residual mass, and radiotherapy was commenced to the lumbar vertebrae 3, 4 and 5. Six months after commencement of treatment, imaging demonstrated a persistent residual mass and biopsy confirmed the existence of residual tumor. Further debulking surgery was performed on the residual tumor.

Autologous peripheral hematopoietic stem cell rescue was performed following conditioning therapy (Table 3). At 8 months post stem cell rescue, the patient had pain and paraesthesia in the right thigh and had developed a limp. Imaging showed this mass to be filling L4 and 5 and obscuring the nerve root. The patient refused further surgery. 24 months after initial diagnosis, the patient developed pulmonary metastasis and died several months later. Subsequent immunohistochemistry for NUTM1 at review showed positive nuclear staining (Fig. 5c).

#### Case summary

The five patients who had NUTM1-positive tumors were all born in Australia. The only one of the five patients with a family history of cancer was Patient 11, who had a maternal aunt with breast cancer, and the patient’s mother also subsequently developed breast carcinoma. Prior to diagnosis, there was no relevant previous history in Patient 11. All five patients with NUTM1-positive tumors died from disease progression. The median time from diagnosis to death from disease within the overall group of undifferentiated tumors was 12 months (4.5 months to 6.2 years) but the median time from diagnosis to death for the NUTM1-positive group was 8 months (4.5 months to 2.4 years).

### Summary of the histology and genetics of NC cases

The histology of the NC cases varied, one had a phenotype similar to a primitive neuroectodermal tumor (PNET), and most were highly cellular and poorly differentiated. Not all showed morphological evidence of squamous differentiation, but when present, this was often focal and abrupt as previously described [1, 13]. Extensive necrosis was common, sometimes with a prominent neutrophil infiltrate. From Patient 11 there was very little material available, which revealed only a very cellular infiltrative morphology, reminiscent of Ewings Sarcoma (see Fig. 5).

NUTM1 immunohistochemistry positivity typically had a speckled nuclear appearance, was strongest in poorly differentiated areas, and was minimal to absent in the squamous differentiated areas (e.g. Figure 1c, where the paler areas with reduced cellularity and negatively stained nuclei represent tumor cells with obvious squamous differentiation). All cases were positive, except Case 4, where only B5-fixed material was available (Fig. 3c). Cytokeratin staining was typically very focal (e.g. Figure 4d), with only an occasional positive cell, or was completely absent (see Fig. 5d).

The cytogenetic translocation was identified in four cases, however, the archival material often yielded metaphases of poor quality. For Patient 3, a normal karyotype was identified, probably reflecting stromal overgrowth. The tumor material did not allow delineation of the exact translocation breakpoints, but we managed to establish tumor cell lines from three patients (Table 2) which has facilitated more detailed molecular genetic analyses [3, 7–9, 25]. The PER-403 cell line was developed from Patient 2, the first reported case of a translocation t(15;19)(q14;p13.1) [3]. From Patients 4 and 6 we established PER-704 [8] and PER-624 [7], respectively. Each of these three lines carries a *BRD4–NUT* translocation as confirmed by RT-PCR, transcriptome sequencing, immunoblot, and immunohistochemistry, although they vary in regard to exact *BRD4* breakpoint position.

## Discussion

NC represents a particularly aggressive tumor with a median survival of less than 7 months. Originally it was thought to be a childhood disease, but NC has now been identified in patients ranging from newborn to 78 years [1, 2]. Since NC is rare, lacks distinct histological features, and is not confined to a particular organ, it is often misdiagnosed. This study aimed to determine the incidence of NC in pediatric and adolescent patients and to highlight the challenges associated with the diagnosis of NC. Our survey was conducted over a 26-year period, and was limited to cases identified as undifferentiated sarcoma or carcinoma diagnosed at a single institution in Western Australia. The population of this state is clearly defined, with large distances to other states. The cancer treatment for children and adolescents is centralised, so the ascertainment for the population of around 2 million is likely to be complete. We were able to find five cases of this rare and aggressive tumor during the reviewed period. No familially-related cases were recognised, but one patient had a family history of breast cancer. Three of the cases were flagged as being t(15;19) translocation carcinomas, or as NC, at the time the patients were diagnosed, and two others were discovered during this review. The numbers confirm that NC is rare. We have calculated an estimated rate of around 0.41 per million years at risk for children (0–16 years). Although there may be some factor that makes Western Australia a high risk for childhood NC, it is most likely that this high ascertainment relates to the practice at PCH of routinely performing cytogenetic analyses of pediatric tumors in Western Australia. The issue of under-recognition, varying by location, is also suggested by the review of Bauer et al. [18], with six cases reported in Massachussets (population 6.5 million in 2010), but only three in the much larger state of California (population 37 million in 2010).

Four patients presented with tumors in the airways and one in the spine, the latter being unusual. Distant bony metastases were seen in two cases, but all cases were highly aggressive. NPC represents a third of all cancers in the upper airways in children and adolescents, and this is a main differential diagnosis of NC. Most of these are not NCs and are associated with Epstein Barr Virus [28, 29]. They are usually not resectable and have some local spread. Despite this, the prognosis for non-NC NPC is now much better. In a recent study, the 5-year overall survival was 80.9%, the progression-free survival was 79.3%, and five of the reported patients had distant metastatic disease [30]. Since this is very different to the poorer outcome seen in NC, suspected NPC tumors should be routinely screened for NUTM1 positivity to rule out a diagnosis of NC.

In the present study, Patient 11 had the longest duration of remission. The tumor was initially diagnosed as a para-spinal PNET, but the cytogenetic analysis showed a t(15;19)(q13;q13.3) translocation, and the tumor was positive for the NUTM1 protein on review. No cell line was derived from this patient and so we were unable to investigate the nature of the fusion. Recent reports [26, 27] of *CIC-NUTM1* translocations are consistent with this patient’s karyotype however and this case may thus represent this newly reported entity. Interestingly, there is another patient described in the literature with a PNET in the iliac bone that was t(15;19) positive, and this patient is known to have the longest survival published to date [20].

The differential diagnosis of a malignant undifferentiated tumor in childhood that shows some evidence of epithelial differentiation by immunohistochemistry is wide and includes PNET, rhabdomyosarcoma—which may occasionally show cytokeratin positivity [31]—rhabdoid tumor, epithelioid sarcoma, desmoplastic small round cell tumor [32], synoval sarcoma, and germ cell tumor. Over the past five decades, the number of tumor entities has grown enormously, and tumors are now classified by their clinical presentation, morphology, immunohistochemistry, as well as molecular studies. NUTM1-positive carcinoma has been found increasingly, and is now described in all ages [17, 18].

The poor clinical outcome of NC patients has prompted studies to determine whether the timing of standard therapy may influence the duration of remission. In a review of 63 patients diagnosed with NC, longer survival times were seen in those who were treated with early radiation therapy and had good surgical resection of the tumor [18]. The median overall survival for patients with NC was 6.7 months and was not related to histology, sex, location or lymph node involvement. The chemotherapy protocol was not shown to make any difference. In the present study, three of the patients with NC did have early radiation as part of their therapy, however, this did not appear to affect their duration of remission.

Given the dismal outcome for NC, it is urgent to define better treatment options for patients. The search for targeted therapies is severely hampered because NC is rare, and model systems are limited. In this context, cell lines grown from NC tumor samples play a critical role in helping to elucidate the biology of this disease and the identification of potential treatments [3, 7–9, 11, 15, 16, 25]. Two classes of drugs have been found to show activity against NC. They include direct inhibitors of the BRD4 portion of the fusion protein, termed BET inhibitors (iBETs) [1, 33–37]. The others are histone deacetylase inhibitors (HDACs), which promote chromatin acetylation in NC cells, resulting in squamous differentiation and in vitro growth arrest [1, 33]. Novel iBET drugs are currently in clinical development and are demonstrating anti-NC activity, with the possibility of integrating these drugs in combination schedules [38].

In two previous investigations, the NC cell lines established from patients in this and other studies were subjected to systematic screening of established and novel antitumor agents [8, 25]. The results indicated that microtubule inhibitors, topoisomerase inhibitors, anthracyclines, and the CDK9 inhibitor flavopiridol are highly cytotoxic in NC cell lines. An extensive screening of kinase inhibitors in a large panel of tumor cell lines also identified CDK9 inhibitors as being particularly cytotoxic for NC cells [39]. We previously reported the response of NC cells to iBETs to vary considerably [25]; the cytotoxic effect of iBETs was an order of magnitude higher in cell lines with *BRD4-NUTM1* (exon11:exon2) translocations compared to those with other *BRD4-NUTM1* translocation variants. The data demonstrated that beside the translocation breakpoint, other biological signals in the genome contribute to the drug response in NC cells, suggesting that therapy options for patients with NC should take the molecular genetic features of each tumor into account.

## Conclusion

In summary, NC is a rare and aggressive tumor that can be difficult to diagnose. This 26-year survey in a geographically isolated state with a well-defined population, allowed us to determined an estimated incidence of NC of around 0.41 per million child years (0–16 yrs. of age) at risk. Our survey of pediatric patients with undifferentiated and difficult-to-categorise malignant tumors supports the contention that a significant number of children with NC in the past may have been misclassified. Immunohistochemistry for NUTM1 is clearly indicated in the assessment of a poorly differentiated pediatric tumor to confirm diagnosis, as well as the analysis of other genetic features to allow better counselling and consideration of novel treatment options.

## Data Availability

All data generated and analyzed during this study are included in the submitted article, hence there are no additional datasets that could be made available.

## Abbreviations

NUT/NUTM1: Nuclear Protein in Testis
NC: NUT carcinoma
NMC: NUT midline carcinoma
PCH: Perth Children’s Hospital
COG: Children’s Oncology Group
H&E: Hematoxylin and eosin
NSE: Neuron-specific enolase
NPC: Nasopharyngeal carcinomas
PPB: Pleuropulmonary blastoma
MPNST: Malignant peripheral nerve sheath tumor
CCG: Children’s Cancer Group
CT: Computed axial tomography
IFRT: Involved field radiation therapy
FISH: Fluorescence in situ hybridization
MRI: Magnetic resonance imaging
PNET: Primitive neuroectodermal tumor
iBET: BET inhibitor
HDAC: Histone deacetylase inhibitor
